# Mr. Humby, on Vaccine Inoculation

**Published:** 1801-07

**Authors:** W. Humby

**Affiliations:** St. Alban's Street


### *To the Editors of the Medical and Phyſical Journal.*

GENTLEMEN,

IN your laſt Journal I obſerved Mr. Philips's Statement of irregularity in the Vaccine Diſeaſe. It is a circumſtance which has frequently occurred to me, and muſt likewiſe to every experienced practitioner in the new inoculation; with reſpect to the inoculation with Variola after the ſubject has paſſed the Vaccine, no doubt, I believe, now remains of the unſuſceptibility of the conſtitution receiving infection; but that the local diſeaſe, occaſioned by Variolous inoculation, after having paſſed the Vaccine, is capable of producing conſtitutional diſeaſe in another ſubject, is a matter not ſo frequently canvaſſed, but which is equally certain. In ſupport of which poſition I offer you the following facts; if you conceive they may be the means of convincing one incredulous friend, they are at your ſervice for publication. I am, &c.

*St. Alban's Street, May* 9, 1801. W. HUMBY.

---

THE beginning of October laſt, I inoculated the infant daughter (three weeks old) of my brother, with Vaccine virus; ſhe paſſed the diſeaſe in the regular way, without indiſpoſition. About two months ſince I heard the mother was averſe to taking the child to a friend's houſe where the Small-pox was; I immediately deſired I might inoculate it with Variola, ſo as to reduce the child's ſafety to a poſitive certainty. I inoculated her on Friday, May the 8th, from a ſubject who had Small-pox from inoculation. The next day, Saturday, the arm arm was inflamed. Sunday, a limpid veſication had riſen. *Monday*, the inflammation began to ſubſide; and on the ninth day, when the child ſhould have had conſtitutional diſeaſe, the eſchar had fallen off, and left a mark as is the caſe in inoculated Small-pox. To convince the mother ſtill farther, on the *Monday* I inoculated two children, (brother and ſiſter) the boy with fluid from her child's arm, the girl with Variola; the girl had the diſtinct Small-pox, though rather full; the boy's arm, on the fourth day after inoculation, had no appearance of infection; but on the fifth day a trifling inflammation was viſible, which went on increaſing till the twelfth, when he ſickened, and had the Small-pox of the confluent kind, which he paſſed ſafely through.

